# Treatment Outcomes of Primary Molars Direct Pulp Capping after 20 Months: A Randomized Controlled Trial

**Published:** 2013-10-07

**Authors:** Masoud Fallahinejad Ghajari, Tahereh Asgharian Jeddi, Sonay Iri, Saeed Asgary

**Affiliations:** a Dental Research Center, Research Institute of Dental sciences, Shahid Beheshti University of Medical Sciences, Tehran, Iran;; b Pediatric Dentistry Department, Dental School, Shahid Beheshti University of Medical Sciences, Tehran, Iran;; c Iranian Center for Endodontic Research, Research Institute of Dental sciences, Shahid Beheshti University of Medical Sciences, Tehran, Iran

**Keywords:** Calcium Enriched Mixture, CEM cement, Deciduous tooth, MTA, Pulp Capping

## Abstract

**Introduction:**

The aim of this randomized controlled trial was to compare the radiographic and clinical success rates of direct pulp capping (DPC) using ProRoot mineral trioxide aggregate (MTA) or calcium enriched mixture (CEM).

**Methods and Materials:**

A total of 42 symptom-free carious vital primary molars (21 pairs) were selected in this split mouth trial and randomly pulpotomized in two experimental groups. Pinpoint pulp exposures were covered by the same blinded operator with MTA or CEM, and then restored by amalgam. Radiographic and clinical successes were evaluated at 20 month follow-up. Data were statistically analyzed using McNemar test.

**Results:**

Nineteen patients were available for 20-month follow-up; only one failed tooth was extracted in the CEM group. All available teeth were symptom-free, however, the final evaluated success rate was 89% in CEM (CI 95%: 0.82-0.96) and 95% in MTA (CI 95%: 0.85-1) groups without statistical difference (*P*=0.360). Worst case scenario was applied for missing value analysis; assuming that the 2 lost cases in CEM group had failed and the only lost case in MTA group was due to treatment success, as a result the success of CEM and MTA were 81% (CI 95%: 0.72-0.90) and 95% (CI 95%:0.85-1), respectively, with no statistical difference (*P*=0.078). In the reverse scenario, the success of MTA and CEM were 86% (CI 95%: 0.78-0.94) and 90% (CI 95%: 0.82-0.98), respectively; again with no statistical difference (*P*=0.479).

**Conclusion:**

Effectiveness of MTA and CEM biomaterials for primary molars’ DPC was similar; CEM can be a suitable alternative for MTA.

## Introduction

Direct pulp capping (DPC) is more common for human permanent teeth rather than deciduous ones [1]. While DPC on immature permanent teeth is a universally accepted treatment protocol, DPC in primary teeth is currently controversial. Reports of favorable pulp response in primary molars after DPC of traumatic or mechanical exposure are rather infrequent; however, positive outcomes for DPC of carious pulp exposure that it is surrounded by normal dentine have been reported [2, 3].

Numerous materials have been suggested for DPC including calcium hydroxide (CH), zinc oxide eugenol (ZOE), formocresol (FC), polycarboxylate, adhesive resins, enamel matrix derivate (EMD), beta-tricalcium phosphate, NaOCl and mineral trioxide aggregate (MTA) [1, 4-7]. As the traditional pulp capping agent, CH showed worse clinical and histological outcomes in comparison with some other tested materials [7-9]; however, a recent clinical trial revealed that DPC with CH or MTA has similar results [10].

MTA is originated from Portland cement and has shown superior sealability and biocompatibility but less cytotoxicity than other pulp covering materials [11]. There are more than 1000 research articles regarding this rather new biomaterial in PubMed [12] which revealed that MTA stimulates the healing of dental pulp and periodontium [13, 14]. Despite being suggested as a suitable alternative for formocresol (FC) in primary premolars pulpotomy [15], it is rather expensive and has a long setting time with potential tooth discoloration [16]. Providentially, introduction of new biomaterials has caused a change in the old idea that DPC of a carious pulp exposure in a primary tooth is not recommended [17-20].

Calcium enriched mixture (CEM) cement demonstrated biocompatibility in both *ex vivo* and *in vivo* studies [21-26]; it has antimicrobial activity, appropriate sealing ability, and quickly sets in aqueous environment [27-29]. Recent randomized clinical trials revealed that CEM pulpotomy in primary molars was equally successful to MTA after 2-year [30, 31]. A recent case report, as the best histological and cone beam computed tomography (CBCT) evidence, showed that CEM can induce a thick and complete calcific bridge with tubular dentin after pulpotomy of a primary molar [32].

We previously reported that DPC of primary molars with MTA or CEM cement at 6-month had similar success rates [33]. The aim of this part of the clinical trial was to assess the long-term treatment outcomes after a 20-month follow up.

## Methods and Materials

This split mouth randomized clinical trial was approved by Ethics Committee of Research Institute of Dental Sciences, Shahid Beheshti University of Medical Sciences, Tehran, Iran and was in compliance with the ethical principles of The Helsinki Declaration.

Same as previously reported materials and methods [33]; twenty one healthy children (5-8 years-old) with at least two carious second primary molars were included based on symptom-free vital pulp exposure, presence of at least two-thirds of the root length, being restorable with amalgam, availability for 20 months follow-up and parents’ acceptance with the informed consent. Exclusion criteria were presence of spontaneous pain, tenderness to percussion, sinus tract, internal/external root resorption, apical/furcation lesion, periodontal pocket >3 mm, pathologic luxation, and absence of successor permanent tooth.

The molars were randomly assigned to experimental groups. The single operator and also the patients were blind to biomaterial/treatment. Each tooth was anesthetized and after isolation, carious lesion was completely removed. Exposed dental pulp was irrigated with normal saline and after achieving the hemostasis it was randomly allocated to be covered either with ProRoot MTA (Dentsply, Tulsa, OK, USA) or CEM (BioniquDent, Tehran, Iran). The teeth were restored by amalgam. Any co-interventions was avoided.

Treatment outcomes based on previously reported criteria [33] were evaluated at 20 months by a calibrated dentist, radiologist and a statistician who were also blind to the type of used biomaterial. Data analysis was performed using the SPSS software, version 16.0 using McNemar test. The Missing Value analysis as well as intention to treat analysis was also performed.

## Results

Two patients (*n*=4 teeth; ~10% dropout) missed for long-term follow-up, due to family migration. Nineteen available patients were evaluated at 20-months. One tooth in CEM group was extracted due to failure and as a result 18 teeth in CEM and 19 teeth in MTA group were available for final assessment. One more teeth in either group also failed; so the per protocol success in CEM and MTA groups were 89% (CI 95%: 0.82-0.96) and 95% (CI 95%: 0.85-1), respectively, without any statistical difference (*P*=0.360). In addition, *intention to treat analysis* showed that there was no significant difference between two groups (*P*=0.417).

*Missing Value analysis* for two opposite worst case scenarios demonstrated that if the two lost cases in CEM group are assumed as failure and the only lost case in MTA group is classified as success, the success rates of CEM and MTA calculated would be 81% (CI 95%: 0.72-0.90) and 95% (CI 95%:0.85-1), respectively, with no difference (*P*=0.078). In opposite scenario the success of MTA and CEM were 86% and 90% (CI 95%: 0.78-0.94 and 0.82-0.98, respectively); again with no statistical difference (*P*=0.479). Therefore, in the two worst case scenarios, the obtained results were comparable and missing data did not affect the outcomes.

## Discussion

This split mouth randomized clinical trial in primary molars with two MTA and CEM endodontic biomaterials is unique as the study protocol was quadruple-blind and the patients’ variability was minimized by employing a split-mouth model. DPC was performed blindly at care-provider/patient level even when the biomaterial was capped; as the appearance of the two pulp capping biomaterials was tooth-colored, the single operator did not know what biomaterial was placed. A blinded pedodontist evaluated the clinical symptoms and radiographic assessments were carried out by a blinded oral radiologist. The statistical analyzing of the data was also carried out blindly. So a well-designed quadruple-blinded randomized clinical trial without researcher’s cognitive bias was carried out. Obtained results revealed that favorable clinical/radiographic treatment outcomes of MTA and CEM cement for primary molars’ DPC were comparable.

Currently, in the common school of thought, DPC for primary teeth is not generally recommended as the previous reported prognosis was not satisfactory [34]; and it is hypothesized that the high cellular content of primary pulp tissue may be responsible for failures via differentiation of mesenchymal cells to odontoclasts that can lead to internal resorption [35]. Our obtained results, however, did not show such cases of failure due to internal resorption. Moreover, our previous report for 6-month follow-up [33] as well as 2-year results of Tuna and Olmez [10] have confirmed the present results and simultaneously reject the hypothesis. It can be hypothesized that under the circumstances of the underlying pulp, inflammatory mediators can trigger differentiation of the mesenchymal cells to odontoclasts which are responsible for dentin resorbtion and thus by shifting the status from inflammatory to reparative, CEM/MTA can overcome this issue.

In recent decade, several well-designed randomized clinical trials as the best current evidence, have assessed the treatment outcomes of MTA versus FC pulpotomy of primary molars; a few systematic reviews in this regard [15, 36, 37] summarized the published evidence and indicated that MTA is superior to FC in primary molars pulpotomy resulting in a lower failure rate. In addition, it was reported that MTA induces a less undesirable response. Therefore the new school of thought considered MTA as the gold standard in primary teeth pulp therapy and we employed this biomaterial for DPC of primary molars as control. Our favorable obtained results for MTA were in accordance with previously reported evidence.

CEM cement has been introduced as a new endodontic biomaterial with different chemical composition from MTA and this trial intended to study the treatment outcomes of DPC with this new biomaterial. Evidence-based success in various vital pulp therapies in human subjects using CEM cement has been documented [28, 38]; recent randomized clinical trials have demonstrated successful treatment outcomes following DPC and pulpotomy of primary molar teeth using CEM cement [30]. Numerous studies have also confirmed that CEM cement as an endodontic sealant is nontoxic [22, 39, 40], biocompatible [41] and promotes dentinogenesis, cementogenesis and osteogenesis when it is in contact with the dental pulp, periradicular tissues or bone, respectively [14, 27, 32, 42]. A recent histological and CBCT evaluation of a human primary molar pulpotomy using CEM demonstrated thick/complete tubular dentin bridge formation [32]. The high success rate of DPC with CEM in the present report is remarkable, particularly considering the 20-month follow-up period which is concurring with previous favorable results.

Dropout rate or missing data can negatively influence the reported effectiveness of randomized clinical trials. In the present report, in addition to intention to treat analysis, we present an alternative missing value analyses using worst case scenario in order to adjust the results for missing values. The analyses revealed that the effectiveness DPC with CEM is so strong that even imputing the worst case scenario did not change the positive results; in other words, missing response rate is so low that it did not modify the results.

## Conclusion

The newer biomaterial, CEM, demonstrated favorable clinical and radiographic successes as a DPC agent in primary molars; it seems to be a suitable alternative for high-price MTA.
